# Geographic variations in access and utilization of cancer screening services: examining disparities among American Indian and Alaska Native Elders

**DOI:** 10.1186/1476-072X-13-18

**Published:** 2014-06-09

**Authors:** Samuel D Towne, Matthew Lee Smith, Marcia G Ory

**Affiliations:** 1 1266 TAMU, Department of Health Promotion and Community Health Sciences, School of Rural Public Health, Texas A&M Health Science Center, College Station, TX 77843-1266, USA; 2 Department of Health Promotion and Behavior, College of Public Health, The University of Georgia, 330 River Road, 315 Ramsey Center, Athens, GA 30602, USA

**Keywords:** American Indian, Alaska Native, Cancer, Rural, Disparities, Ecological analysis

## Abstract

**Background:**

Despite recommendations for cancer screening for breast and colorectal cancer among the Medicare population, preventive screenings rates are often lower among vulnerable populations such as the small but rapidly growing older American Indian and Alaska Native (AIAN) population. This study seeks to identify potential disparities in the availability of screening services, distance to care, and the utilization of cancer screening services for Medicare beneficiaries residing in areas with a higher concentration of AIAN populations.

**Methods:**

Using the county (n =3,225) as the level of analysis, we conducted a cross-sectional analysis of RTI International’s Spatial Impact Factor Data (2012) to determine the level of disparities for AIAN individuals. The outcomes of interest include: the presence of health care facilities in the county, the average distance in miles to the closest provider of mammography and colonoscopy (analyzed separately) and utilization of screening services (percent of adults aged 65 and older screened by county).

**Results:**

Counties with higher concentrations of AIAN individuals had greater disparities in access and utilization of cancer screening services. Even after adjusting for income, education, state of residence, population 65 and older and rurality, areas with higher levels of AIAN individuals were more likely to see disparities with regard to health care services related to mammograms (p ≤ .05; longer distance, lower screening) and colonoscopies (p ≤ .05; longer distance, lower screening).

**Conclusions:**

These findings provide evidence of a gap in service availability, utilization and access facing areas with higher levels of AIAN individuals throughout the US. Without adequate resources in place, these areas will continue to have less access to services and poorer health which will be accelerated as the population of older adults grows.

## Background

According to the United Nations, there are over 370 million native or indigenous peoples residing in approximately 90 countries [1]. In the United States (US), as of the 2010 Census, there were approximately 5.2 million American Indian or Alaska Native (AIAN) individuals, which is a growth of almost 25% over 10 years [2,3]. This population is expected to reach 8.6 million by 2050 [4]. At the same time, the AIAN population aged 60 and older is expected to increase from just over 600,000 in 2010 to approximately 1.8 million in 2050 [5].

The United States Preventive Services Task Force (USPSTF) recommendation for female adults aged 65 and older (up to 75) includes having a biennial mammogram to screen for breast cancer [6]. The USPSTF also recommends screening for those aged 50 and older (up to 75) for colorectal cancer using fecal occult blood testing (every 5 years), sigmoidoscopy (every 5 years), or colonoscopy (every 10 years) [7]. The existing literature for AIAN individuals is sparse relative to other groups, especially related to cancer screening among older AIAN adults.

The diagnosis of late stage cancer is a predictor of lower survival rates in those diagnosed [8]. In 1987, an estimated 18.6% of American Indian (AI) women aged 50-59 had reported receiving a mammogram [9]. More recent estimates (e.g. California in 2001) indicate gaps in screening in the last two years among AIAN women, as compared to White women, this gap persists among those with incomes below 200% of the federal poverty level, at 61% of AIAN individuals lower than White (71.6%); Latino (66.5%); Asian (63.2%) and African American individuals (72.8%) [10].

Nationally (rates calculated from the Behavioral Risk Factor Surveillance System [BRFSS] 1999-2002 and 2004), the percent of female adults aged 40 and older with mammography in the past two years was 76.2% among non-Hispanic White adults and 69.0% among AIAN adults [11]. The rates of women aged 40 and older receiving a mammogram within the past 2 years were lowest for AIAN adults (72.8% in 2005; 62.7% in 2008); as compared to White adults (67.4% in 2005; 67.9% in 2008); Black adults (64.9% in 2005; 68.0% in 2008), and Asians adults (54.6% in 2005; 66.1% in 2008) [12]. Here, comparisons within all other races including Hispanic or Latina ethnicities shows an increase in the rates of receiving a mammogram from 2005 to 2008, that is, all but for AIAN individuals [12]. Additionally, the age-adjusted cancer incidence rates for all cancer sites across all races dropped from 1990 to 2004, except for AIAN individuals [12]. The incidence rate of invasive female breast cancer among AIAN adults was highest among those aged 65 and older from 1999-2004 [13]. A small study (n = 550) in Washington state found rates of having ever received a mammogram at 58% for AIAN adults aged 65 and older [14].

Nationally, the rate of colorectal cancer screening (endoscopy in the past 5 years) in adults aged 50 and older was 43.7% and 39.5% among non-Hispanic White adults and 36.5% and 36.2% among AIAN adults for males and females respectively (rates calculated with BRFSS using 1999-2002 and 2004) [11]. Colorectal cancer being diagnosed in the early stages (local) was measured at lower rates for AIAN adults aged 50 and older as compared to non-Hispanic White adults; at 62.2 versus 46.9 (per 100,000) for the years 2001-2004 [11], p.2140, Table nine. In Washington State, the rate of ever receiving colorectal cancer screening (fecal occult blood) was approximately 46% among AIAN adults aged 65 and older in a small study with 550 AIAN adults [14]. Colorectal cancer cases among those aged 65 and older accounted for approximately 65% of all cases from 1999-2004 among AIAN individuals [15].

As a follow-up to screening disparities, another area of concern is that of having a usual source of care where one may receive these screening services. Indian Health Service provides primary care for just 1.9 million of the 4.3 million AIAN adults residing in the US [16,17]. This suggests a potentially severe gap in available services to over half of AIAN individuals residing in the US. The combination of residents residing in remote rural areas and the low-level of funding to Indian Health Service has contributed to this gap in services [11]. The coverage from IHS varies across tribes and not all AIAN adults may qualify for IHS coverage [18]. In 2006, over 90% of AIAN older adults (age 65 and older) were enrolled in Medicare [19].

As a group, AIAN individuals rank low on many social and environmental indicators of health and those related to access and utilization of health care services. Nationally, over 26% of the American Indian population is living below the federal poverty level [20], and rates of poverty for AIAN individuals are three times higher than those reported for non-Hispanic White individuals [11]. AIAN individuals are more likely than other racial groups in the US to have lower educational attainment and face economic hardships [21,22]. Additionally, AIAN adults aged 65 and older were more likely to report having no health insurance compared to their non-Hispanic White counterparts (4.1% compared to 1.5%) (BRFSS data 1999-2002 and 2004-2005) [21].

These social determinants of health (high poverty and lower education) increase the risk of having poor access to health care services [23], including cancer screening services. This is especially true for older adults who may have more barriers (psychological and physical) in access to health care services (e.g. transportation, disabilities, distance, perceived barriers) [24,25]. Contextual factors including environmental characteristics (i.e. distance to resources such as food or health care services) of one’s community or the socio-economic characteristics of one’s neighborhood or working environment play a key role in one’s overall health behaviors [26,27].

A small study of rural AIAN adults identified lack of access to medical personnel and long travel distances as barriers to accessing cancer screening [28]. Rural areas also suffer from disproportionate gaps in health care services, where rural residents also suffer disproportionate disability and disease when compared to their urban counterparts [29-34]. Additionally, a majority of AIAN individuals live in urban areas, where Indian Health Services has a limited infrastructure in place with highly variable health care services [35], p5.

Thus, the combination of social determinants of health and older age and infrastructure of one’s environment compound the risk impacting AIAN older adults. The relative gap in research on this vulnerable population provides compelling evidence of the need to understand more about the AIAN older adult experience with regard to accessing and utilizing health care services. Furthermore, older AIAN individuals represent a vulnerable and understudied group in which the cancer burden is expected to grow [36].

### Objectives

This study sought to identify potential disparities in the availability and accessibility of health care services and the utilization of screening services for AIAN individuals aged 65 and older. We used county-level analyses that takes aggregate data across the US and identifies whether there are gaps in available service, access to providers and utilization of cancer screening services in areas with a higher proportion of AIAN individuals, which overlap with rural areas in many cases. We had three overall objectives in the current study. First, we measured the overall rates of unmet breast and colorectal cancer screening among AIAN older adults. Second, we identified socio-demographic characteristic of areas with a higher concentration of AIAN individuals. Third, we identified whether disparities were present with regard to geographic barriers in accessing cancer screening services. Furthermore, this study examined whether individuals likely to face these geographic barriers were also likely to have lower screening rates than those in other areas.

## Methods

### Data source and target population

We conducted a cross-sectional analysis of Research Triangle Institute (RTI) International’s Spatial Impact Factor database (version 5, May 2012). The unit of analysis was the county (n = 3,225). This included all counties within the US. The target population was the resident population in areas with higher proportions of AIAN individuals compared to all other areas. The proportion of AIAN residents was separated into 2 levels across two separate variables. The first variable was split as follows: *high* defined as above the average at 1.87% (n = 370) versus at/below the average in 2006 by county. The second variable split was another two-way dichotomization where areas were separated into *very high* defined as at/above the 95^th^ percentile at 7.25% (n = 157) versus all other areas (below the 95^th^ percentile). These percentage splits were based on the proportion of AIAN among the entire county population.

We also used the BRFSS (2010) to measure the overall unmet recommended screening (i.e. never received screening or not received screening within the recommended time-frame) among this population. The BRFSS data was not incorporated in county analysis. Using data from 2010 allows us to provide reasonably current rates of screening among AIAN adults, while at the same time staying within a reasonable timeline (i.e. 4 years) from our analysis of those individuals residing in areas with a greater concentration of AIAN residents. Here, we provide individual-level analysis for AIAN populations by age group as part of our descriptive analysis. The BRFSS data was not incorporated into our geographic analysis of areas with higher levels of AIAN individuals, but is provided as a national snapshot of unmet recommended screening among AIAN adults. The BRFSS data was restricted to the non-institutionalized adult population participating in the BRFSS annual landline telephone survey and is nationally (US) representative. More information on the BRFSS methodology and limitations can be found on the Centers for Disease Control and Prevention’s website (http://www.cdc.gov/brfss/).

Our sample size for the 2010 BRFSS data included 2,507,111 for non-Hispanic AIAN adults and 161,180,359 for non-Hispanic White adults. After restricting to those aged 65 and older, our sample size was further reduced to 311,032 for non-Hispanic AIAN adults and 32,703,850 for non-Hispanic White adults.

### Outcome variables

The outcomes of interest included the number of unique cancer screening providers, utilization of screening procedures, and distance from providers for the residential population of interest. The number of healthcare facilities by county was calculated from 2006 data (most current available public use file for our measures) reported by the Centers for Medicare and Medicaid Services (CMS) [37]. Provider data was identified as the number of unique cancer screening providers including: mammography providers (mammogram and MRI) and in a separate variable colonoscopy providers identified by UPIN with ZIP Code centroid inside the area in 2006.

Distance to facilities was calculated as the average distance in miles to the closest provider (colonoscopy provider and separately mammography provider) in ZIP Codes with centroid in this geography unit (county) in 2006. This was calculated as the beneficiary population-weighted average distance (miles) over all ZIP Codes with centroid in this geography unit to closest provider ZIP Code.

The utilization of screening services was calculated as the percent of persons with a mammography for females and in a separate variable for the percent of persons with a colonoscopy (males and females) in 2006. The data is based on 100% CMS carrier file claims by procedure codes [37]. Again, we used the most current public use file available from RTI. The percent was based on those Medicare eligible population (i.e. age 65 - 104 years, alive the entire year, with 11-12 months of FFS Part A and B). Using data for those aged 65 and older allowed us to separate out cost as a large predictor of screening among those 65 and older, who typically receive care through Medicare. Data was suppressed for areas with less than 11 persons in the denominator. There were less than 1% of counties with missing values.

### Control variables

Rurality, median household income at the county and education were included as ecological social determinants of health. We used 2013 Urban Influence Codes (UIC) to identify rural separations taken from the United States Department of Agricultures’ Economic Research Services. Rurality was included in the multivariate logistic regression equation and separated into metropolitan (UIC 1-2), micropolitan (UIC 3, 5, 8), small rural (UIC 4, 6, 7) and remote rural (9-12). The UIC classification is based on elements of population size and for non-metropolitan areas, this extends to the relative adjacency to metropolitan areas or not [38]. Income was calculated based on estimates of the median household income for 2005 based on US Census Bureaus’ small area Income and Poverty estimates [37]. Education was based on the proportion of the population aged 25 and older with less than a high school diploma or equivalent. The number of males and females in the county aged 65 and older was included to capture differences in the older population. We also identified states as a measure of differences between/across states. Finally, we included Health Professional Shortage Areas in our descriptive analysis to identify differences across differing levels of AIAN concentration and rurality.

### Statistical analysis

We used bivariate and multivariate regression to assess differences in distance, utilization and availability of cancer screening and cancer screening providers in areas with higher levels of AIAN individuals versus all other areas. Analysis was conducted with SAS 9.4 [39]. Mapping was conducted using ArcGIS 10.2 for Desktop [40].

## Results

For our first objective, we first created a brief individual-level snapshot of the rates of unmet recommended screening among non-Hispanic AIAN and non-Hispanic White adults in 2010. For our second and third objectives, we then provided a snapshot of the intersection of geographic barriers to accessing health care services, while at the same time providing the overall screening rates for breast and colorectal cancer in areas with higher concentrations of AIAN individuals in 2006. In addition, due to the relatively high rural presence in areas of higher concentrations of AIAN individuals, we provide a subgroup analysis of rurality for our screening-related measures. Finally, we provide the results of our regression analysis.

### Descriptive analyses

Individual-level analysis: Our individual-level analysis provides national measures for unmet recommended screening for AIAN individuals. In 2010, the rate of ever having a mammogram was 67% for non-Hispanic AIAN adults as compared to 71% for non-Hispanic White adults. This gap of ever being screened was greater for colonoscopy/sigmoidoscopy, where non-Hispanic AIAN adults had rates of 55% versus 68% for non-Hispanic White adults in 2010.

We restricted analysis to those aged 65 and older to identify rates of unmet screening for non-Hispanic White and non-Hispanic AIAN adults in 2010. The rate of ever receiving a mammogram was similar among AIAN and White adults at 95% and 96% respectively. However, we find that the rate of receiving a mammogram in the past two years was lower for AIAN individuals at 75% as compared to 81% among White adults in 2010. Furthermore, we find that the rate of ever receiving a colonoscopy/sigmoidoscopy was lower among AIAN adults (68%) as compared to White adults (77%). The rate of receiving a colonoscopy/sigmoidoscopy in the past 10 years was similar across these two groups at 94% and 95% for AIAN and White adults, respectively in 2010.

Geographic comparisons: We measured the socio-economic characteristics of areas with higher concentrations of AIAN individuals in order to understand some of the underlying social determinants of health that may impact one’s health-related behaviors. A key social determinant of health, income, was lower in areas with a *high* presence of AIAN individuals. This gap was greater with *very high* proportions of AIAN individuals (i.e. 95^th^ percentile split). In addition, the number of older adults in these areas was substantially lower in areas with a higher proportion of AIAN individuals (see Table 1).

**Table 1:** Population demographics and geographic distribution of areas with a higher presence of AIAN individuals

	**AIAN presence**				**Total**
	**Mean split**		**95**^**th **^**percentile split**		
	**Above 1.87% mean**	**At/below 1.87% mean**	**At/above 95**^**th **^**percentile**	**Below 95**^**th **^**percentile**	
**Household income (2005)**	$36,557.76*	$39,505.66*	$35,322.55*	$39,360.18*	$39,158.30
**Education (proportion aged 25 and older with less than a High school diploma or equivalent)**	20%*	23%*	22%	23%	23%
**Females aged 65 and up**	2,728*	6,959*	1,655*	6,720*	6,473
**Males aged 65 and up**	2,062*	4,855*	1,275*	4,701*	4,534
**Geographic distribution**					
**Urban influence codes (UICs)**					
**Rurality 4-way split**					
Urban (UIC 1-2)	69	1167	17	1219	
Micro (UIC 3,5,8)	82	564	28	618	
Small rural adjacent to metro (UIC 4,6,7)	84	573	37	620	
Remote rural (UIC 9-12)	133	549	73	609	

*significant difference (*p* ≤ .05).

The distribution of areas with a higher concentration of AIAN individuals was reasonably uniform in areas that were urban, micropolitan and small rural, while areas that were remote rural had a greater frequency of these higher concentration areas when compared to less rural areas. However, the majority of high and very high AIAN presence areas were not located in remote rural areas. Approximately 64% of areas defined as *high* AIAN presence were located outside of remote rural areas, while 53% of *very high* (at/above the upper quartile) AIAN presence areas were located outside of remote rural areas (see Table 1).

Overall, the average screening rate for areas with a higher concentration of AIAN individuals was lower than that of areas with a lower concentration of AIAN individuals (see Table 2). This was true for both mammography and colonoscopy in areas with *high* and *very high* levels of AIAN individuals.

**Table 2:** Area Provider Characteristics and Screening Rates across differing levels of AIAN populations, by county

	**AIAN presence**				**Mean (total)**
	**Mean split**		**95**^**th **^**percentile split**		
	**Above 1.87% mean**	**At/Below 1.87% mean**	**At/Above 95**^**th **^**percentile**	**Below 95**^**th **^**percentile**	
**Percent screened**					
Mammography (females)	37.32%*	38.67%*	34.10%*	38.74%*	38.52%
Colonoscopy	8.96%*	9.42%*	8.63%*	9.41%*	9.37%
**Distance to providers (miles)**					
Mammography	31.89*	14.93*	45.02*	15.44*	16.88
Colonoscopy	26.15*	10.90*	38.33*	11.34*	12.66
**Average number of providers**					
Mammography	8.32*	15.31*	4.49*	15.01*	14.51
Colonoscopy	5.92*	12.13*	3.88*	11.79*	11.42
**Health Professional Shortage Areas (HPSA) primary care**					
Whole or partial county	332	2071	142	2261	
Non-HPSA	38	784	15	807	

*significant difference (*p* ≤ .05).

Furthermore, the average distance to providers in areas with a higher presence of AIAN individuals was at least twice that of areas with a lower presence of AIAN individuals. Here, the average distance to a colonoscopy provider was nearly three times that of all other areas (nearly 26 miles versus 11 miles) when comparing areas above the mean (*high presence*), while this distance was nearly 4 times that of all other areas when comparing differing levels based on the 95^th^ percentile (see Table 2). For mammography, the average distance was twice and three times that of all other areas when considering the mean split and the 95^th^ percentile split (*very high presence*) respectively. Here, the average distance to a mammography provider in areas with a *very high* AIAN presence was over 45 miles versus approximately 15 miles in all other areas.

**Figure 1:**
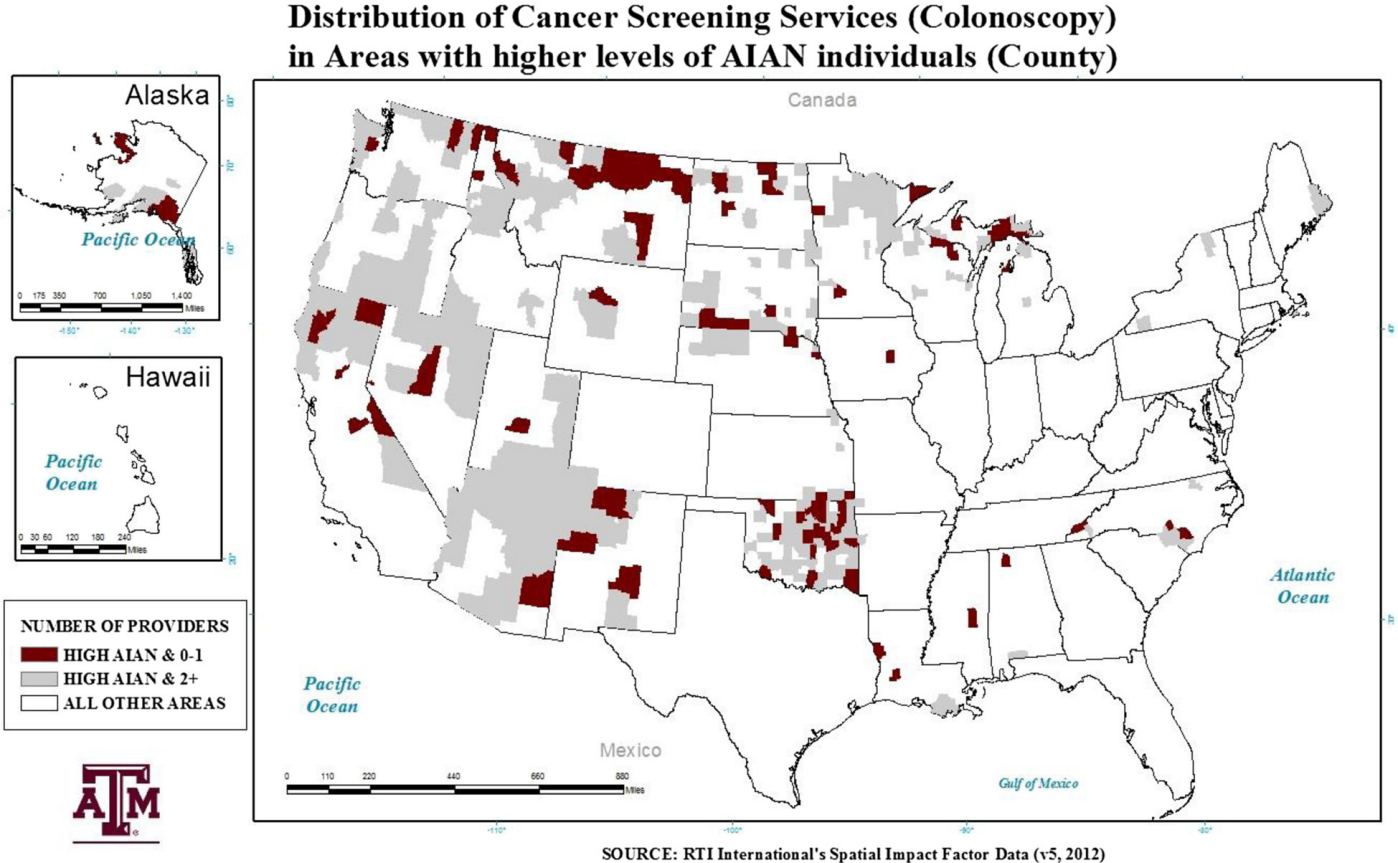
Areas with a high (Above 1.87%) AIAN presence and the distribution of colorectal cancer screening providers.

**Figure 2:**
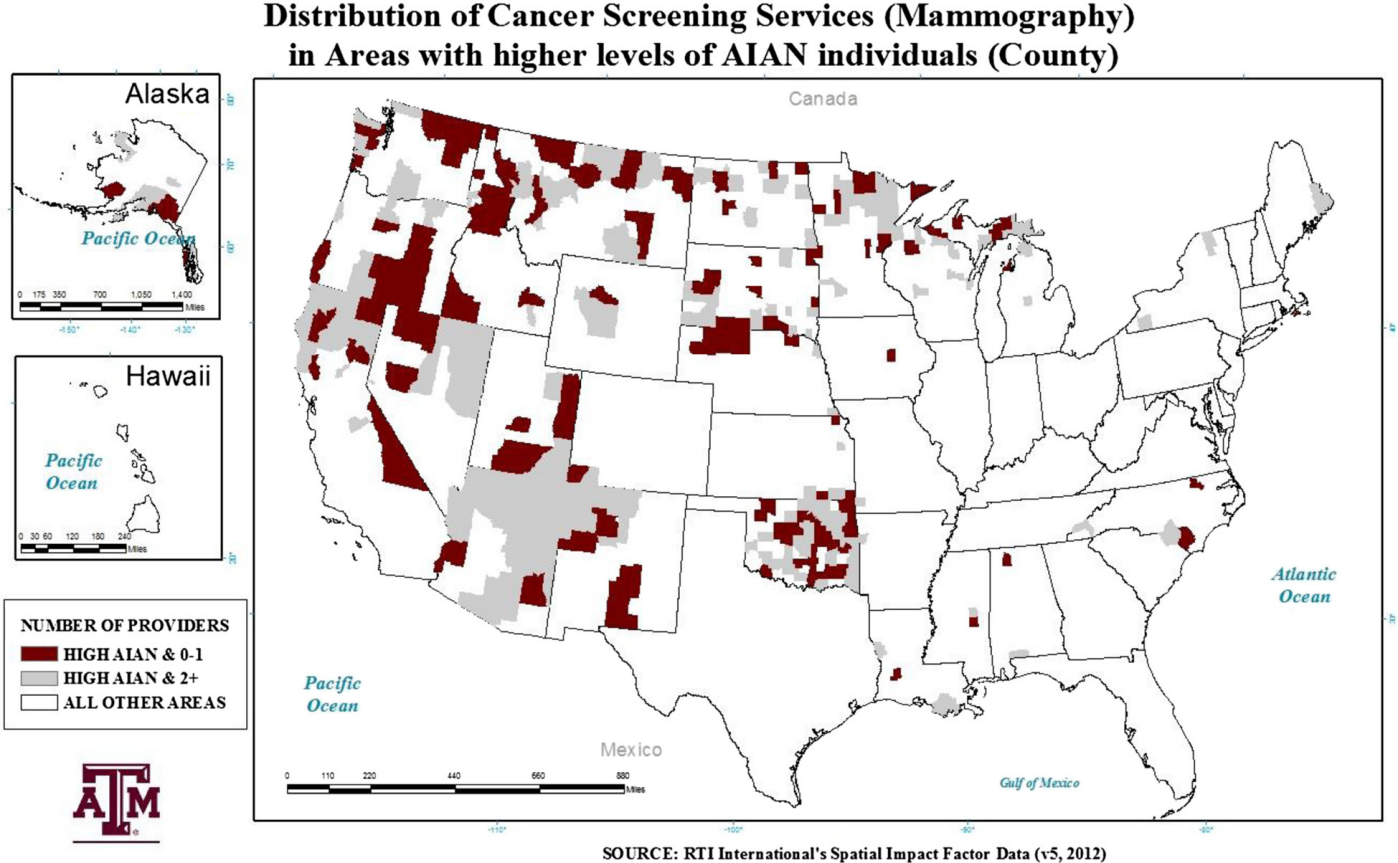
Areas with a high (Above 1.87%) AIAN presence and the distribution of breast cancer screening providers.

The average number of unique providers differed greatly across areas with differing levels of AIAN individuals (see Figures 1 and 2). The average number of providers in areas with a *high* (above the mean) concentration of AIAN individuals was half that of all other areas for colonoscopy providers. Areas with a *very high* (at/above the 95^th^ percentile) presence of AIAN individuals had, on average, less than 5 mammography providers per county and approximately 4 colonoscopy providers per county.

#### Rurality subgroup analyses

Tables 2 and 3 provide the results from distribution analyses about the screening utilization, distance to providers and average number of providers by rurality (see Table 3). Here, rates of screening were lower for more rural areas for both colorectal screening and mammography. In addition, there were greater distances to providers in more rural counties where the average distance to providers among remote rural areas is approximately three times that of urban areas. The average number of providers was drastically different for areas outside of urban counties collectively. Here, the number of providers dropped dramatically from urban areas to all other areas. For the most rural counties, there were approximately two providers for each screening services and six (mammography) or seven (colonoscopy) for areas that were micropolitan. Again, this is the average at the *county*.

**Table 3:** Area provider characteristics and screening rates across differing levels of Rurality, by county

	**Rurality**				**Mean (Total)**
	**Urban (ref.)**	**Micropolitan**	**Small Rural adjacent to metro**	**Remote Rural**	
**Percent screened**					
Mammography (females)	40.09%	39.07%*	37.27%*	36.36%*	38.52%
Colonoscopy	9.70%	9.50%*	9.25%*	8.78%*	9.37%
**Distance to providers (miles)**					
Mammography	9.25	12.90*	18.79*	32.53*	16.88
Colonoscopy	7.63	10.25*	13.56*	23.01*	12.66
**Average number of providers**					
Mammography	28.93	6.63*	2.27*	1.94*	14.51
Colonoscopy	21.71	5.80*	2.64*	2.49*	11.42
**Health Professional Shortage Areas (HPSA) primary care**					
Whole or partial county	866	450	530	550	
Non-HPSA	370	196	127	127	

*significant difference (p ≤ .05).

Note: Rural differences were compared to the referent (ref.) Urban.

### Regression analysis

For regression analyses, we split the percent screened into at/below the lower quartile (34.16% for mammography among females; 8.36% for colonoscopy among males and females) and at/above the upper quartile (20.5 miles for mammography; 14.7 miles for colonoscopy) with regards to distance to providers in the county. We also split the number of providers into 1 or none versus 2 or more per county. This allowed us to calculate Odds Ratios (OR) for the likelihood of our outcomes (see Tables 4 and 5).

**Table 4:** Regression analysis

	**AIAN presence**					
	**Above 1.87% mean**			**At/Above 95th percentile**		
	**Odds ratio**	**95% confidence intervals**		**Odds ratio**	**95% confidence intervals**	
**Screening (At/below lower quartile)**						
Mammography (females)	**1.452**	1.149	1.835	**2.920**	2.112	4.036
Colonoscopy	**1.793**	1.427	2.254	**2.847**	2.059	3.936
**Distance to providers in miles (at/above upper quartile)**						
Mammography	**2.914**	2.332	3.642	**3.567**	2.579	4.932
Colonoscopy	**3.820**	3.058	4.772	**4.446**	3.206	6.167
**Presence of providers (0-1 versus 2+)**						
Mammography	**1.309**	1.016	1.687	**1.564**	1.071	2.283
Colonoscopy	**1.467**	1.110	1.939	**2.404**	1.631	3.544

Bolded indicates significant differences (p ≤ .05).

**Table 5:** Fully-adjusted regression analysis

	**AIAN presence**					
	**Above 1.87% mean**			**At/Above 95th percentile**		
	**Odds ratio**	**95% confidence intervals**		**Odds ratio**	**95% confidence intervals**	
**Screening (at/below lower quartile)**						
Mammography (females)	**1.840**	1.409	2.401	**2.982**	2.081	4.273
Colonoscopy	**1.696**	1.326	2.168	**2.303**	1.638	3.237
**Distance to providers in miles (at/above upper quartile)**						
Mammography	**1.436**	1.072	1.924	1.377	0.919	2.064
Colonoscopy	**2.346**	1.776	3.100	**2.113**	1.432	3.118
**Presence of providers (0-1 versus 2+)**						
Mammography	**0.652**	0.479	0.888	**0.613**	0.399	0.942
Colonoscopy	0.949	0.685	1.315	1.353	0.875	2.092

Bolded indicates significant differences (p ≤ .05).

Note: Adjusted analysis control for state, income, education, population 65 and older and rurality.

The likelihood of having very low screening rates (at/below the lower quartile) differed by AIAN presence. Areas with high levels of AIAN individuals were more likely to have at/below the lower quartile of mammography (females) screening (OR = 1.84) after controlling for all else in the model. Similarly, areas with very high levels of AIAN individuals were also more likely to have at/below the lower quartile for mammography (females) screening (OR = 2.98) than all other areas (see Table 5).

Similar results were identified for colonoscopy. Areas with high and very high levels of AIAN individuals were more likely to have very low screening rates (OR = 1.70 and OR = 2.30 respectively) after controlling for all else in the model (see Table 5).

The likelihood of having very long distances to providers (at/above the upper quartile) also differed by AIAN presence. Areas with a high presence of AIAN individuals were more likely to have very long distances to providers of mammography (OR = 1.44) and colonoscopy (OR = 2.35). The same was true for areas with very high levels of AIAN individuals for distance to colonoscopy providers; however, there was no difference for distance to mammography across these same areas, after controlling for all other terms in the model (see Table 5).

After controlling for all other terms in the model, the likelihood of having 1 or no providers in the county did not indicate a disparity for areas with a higher AIAN presence (see Table 5). Here, the opposite was true except for areas with a very high presence of AIAN individuals with regard to colonoscopy providers (no difference). In contrast, all of these indicators showed evidence of a gap (i.e. more likely in the odds to have one or no providers in the county) in available care in unadjusted analysis (see Table 4).

## Discussion

### Limitations

We were limited to data from 2006, which is the most current public use file available through RTI’s database. However, this data is based on 100% claims data from CMS, which allows us to limit cost and insurance status as a major barrier to care among Medicare beneficiaries. We focus on Medicare beneficiaries for our measures of screening; however, the proportion of AIAN adults that are Medicare beneficiaries in general is relatively low in most counties (ranging from approximately 1%-56% in 2001; and approximately 2%-96% in 2006). The average proportion of AIAN Medicare beneficiaries identified in our analysis more than doubled between 2001 and 2006. Thus, the strength of this analysis is in identifying gaps that exist and are expected to worsen if left alone, as the population of older adults grows.

National studies conducted within the AIAN population rarely identify tribal associations for those identified as either at risk or having disease or disability. If this variable is not included as a possible covariate in national studies unobserved bias may result. Unobserved variations attributable to aggregate grouping of AIAN individuals across the US without identifying tribal variation may provide less accurate estimates of trends in health status. Thus, the current study of AIAN areas is limited by the national aggregation of all tribes and groups across the US.

Aggregate level data at the census tract or county-level has been shown to provide less variation as compared to more micro-level data, thus, less power in the analysis of comparisons across populations [41]. In addition, we did not have individual-level data that may allow for a greater degree of accuracy when measuring the screening rate for different racial and ethnic groups within counties. We cannot make assumptions for individuals’ in counties directly from our aggregate data (i.e. ecological fallacy). However, we include individual-level data from the 2010 BRFSS in our descriptive analysis which provides national rates of screening among non-Hispanic AIAN and non-Hispanic White adults.

#### Interpretation

One’s geographic location and their potential access and availability of cancer screening services and utilization of cancer screening are related. This study provides a snapshot of the level of access, availability, and utilization of health care services for areas with a higher versus lower proportion of AIAN individuals. Individuals in areas with higher concentrations of AIAN individuals have large gaps in the availability, utilization, and distance to providers when compared to other areas. In addition, non-Hispanic AIAN individuals have higher unmet recommended screening than non-Hispanic White adults. These findings compliment earlier studies that measure gaps in screening among AIAN individuals [11]. In addition, Meersman (2009) found similar findings when measuring mammography use among Hispanic populations in relation to density of providers [27]. Here, the authors report mammography utilization was higher in neighborhoods with a greater density of providers. The relative gap in access, availability and utilization in rural areas is also concerning. The measure of rurality used may affect the degree of this gap, as other measures consist of different characteristics (e.g. RUCA) [42].

#### Policy implications

Those at the federal and state levels in congress are urged to work with tribal leaders to close the gap in access and availability for health care for both rural America and AIAN adults, as in many cases the two overlap. Improvements in screening awareness and improvements in the availability of specialists in rural areas continues to be of concern, especially as the population is experiencing the results of the baby-boom generation turning 65 years of age.

Congress funds only 60% of the need in health care for Native Americans [43]. Inadequacies have been reported with regard to budget adjustments for inflation for Indian Health Service budgets [44]. In addition, the current funding by Congress appropriated for IHS is less than half that of the medical spending per capita for the US population [20]. The Indian Health Care Improvement Act was made permanent with the passage of the Patient Protection and Affordable Care Act (ACA) [45]. This piece of legislation is intended to address issues related to improvements in the collection of reimbursement from Medicare and Medicaid, authorizing services for home and community-based services and other general health services-related issues [45]. Ensuring adequate reimbursement to providers and funding for the infrastructure to provide health care services and policies that support these efforts are needed. The realized implications of this legislation remain to be seen.

#### Research and practice implications

Studies that seek to address factors that lessen disparities through increased research within this population will add to the identification of targets for policy-level interventions, public health awareness campaigns, increased community-level support for changing modifiable risk factors for disease and garner more attention and support from congressional and tribal leaders to increase support for these efforts. Additionally, there is a need to understand the diverse groups comprising AIAN individuals. Currently, there are approximately 565 federally recognized Native American (NA) tribes in the US [46]. Health behaviors of AIAN individuals are not assumed to be uniform across each tribe because differences may exist based on the heterogeneous cultural nuances within this population. Investigators conducting research within these communities must take special action to ensure cultural competency is maintained; due to the vast array of different tribes and the rich history and traditions that are unique to these individuals. Health researchers, health care providers, and policy makers must identify the unique needs of individual tribes and regions with added emphasis on training health professionals in culturally appropriate skills needed when interacting with tribes and AIAN individuals moving into the future. Funding and support for this research in addition to having adequate resources in place for health care services in these areas is a must.

Our study is unique in that it concentrates on a population that is understudied, expected to grow rapidly and has many unique cultural traditions that demand a culturally competent health care workforce. Concentrating on the geographic presence of providers and their distance to residents in these areas of high AIAN concentration and rural areas provides a unique perspective for policy makers and providers serving these areas. This work allows policy makers, legislators and providers to take this evidence into practice via identifying strategies that will target awareness and cultural perspectives in seeking care coupled with finding innovative ways to circumnavigate the accessibility gaps facing these individuals.

From an international perspective, native peoples face similar disparities in cancer screening as compared to those in the current study. For example, a study from Canada reported that individuals residing in densely populated native communities were more likely to lack recent (within last ~4 years) cervical cancer screening [47] (p97). Similar findings are reported in Australia, where native or indigenous peoples also face gaps in cancer screening participation and survival [48,49]. These examples illustrate that gaps in the receipt of cancer screening are not solely endemic to the US. Further research is needed to identify strategies for linking native individuals to screening, which could include the use of tailored messages and native community engagement as possible solutions [50,51]. Internationally, researchers should continue to include native individuals in research because their inclusion is essential to identifying screening trends and improving access to potentially life-saving diagnosis throughout our global community.

#### IRB approval

This study was approved by the Texas A&M University Institutional Review Board (IRB) in December 2013 (IRB number: IRB2013-0780M).

## Competing interests

The authors declare that they have no competing interests.

## Authors’ contribution

SDT, participated in developing the concept for the manuscript, drafting and revision of the manuscript, and data analyses and interpretation. MLS, participated in drafting and revision of the manuscript, and developing the concept for the manuscript. MGO, participated in drafting and revision of the manuscript, and developing the concept for the manuscript. All authors read and approved the final manuscript.
